# HIV-1 Coinfection Does Not Reduce Exposure to Rifampin, Isoniazid, and Pyrazinamide in South African Tuberculosis Outpatients

**DOI:** 10.1128/AAC.00480-16

**Published:** 2016-09-23

**Authors:** Neesha Rockwood, Graeme Meintjes, Maxwell Chirehwa, Lubbe Wiesner, Helen McIlleron, Robert J. Wilkinson, Paolo Denti

**Affiliations:** a Department of Medicine, Imperial College, London, United Kingdom; b Clinical Infectious Diseases Research Initiative, Institute of Infectious Disease and Molecular Medicine, University of Cape Town, Cape Town, South Africa; c Division of Clinical Pharmacology, Department of Medicine, University of Cape Town, Cape Town, South Africa; d Department of Medicine, University of Cape Town, Cape Town, South Africa; e Francis Crick Institute Mill Hill Laboratory, London, United Kingdom

## Abstract

There are contrasting data in the literature about antituberculosis plasma drug concentrations in HIV-1-coinfected patients. We report the pharmacokinetics of rifampin, isoniazid, and pyrazinamide in a cohort of patients being treated for active tuberculosis, the majority of whom were coinfected with HIV-1 and had commenced antiretroviral therapy within 2 months of starting antituberculosis treatment. We also examined the association between antituberculosis drug concentrations and reported drug side effects at the 2-month clinical review. One hundred patients with pulmonary tuberculosis (65% coinfected with HIV-1) were intensively sampled to determine rifampin, isoniazid, and pyrazinamide plasma concentrations after 7 to 8 weeks of a daily quadruple-therapy regimen dosed according to World Health Organization (WHO) weight bands. Pharmacokinetic parameters were determined for each patient by using nonlinear mixed-effects models. HIV-1-coinfected patients had lower clearance rates for rifampin (21% decrease) and isoniazid (23% decrease) than HIV-1-uninfected patients, with resulting higher areas under the concentration-time curve from 0 to 24 h (AUC_0–24_) and maximum concentrations of drug in serum (*C*_max_). Antiretroviral therapy (ART) that included double-standard-dose lopinavir/ritonavir further lowered rifampin clearance, by 46%, and increased the AUC_0–24_. The current uniform dosing (per kilogram of body weight) across WHO weight bands was associated with a trend of decreased pharmacokinetic exposures for the lowest weight band. Use of fat-free mass as opposed to total body weight for allometric scaling of clearance significantly improved the model. Ambulant HIV-1-coinfected patients, the majority of whom were coprescribed ART, did not have reduced antituberculosis drug concentrations compared to HIV-1-uninfected patients.

## INTRODUCTION

Despite global initiatives prioritizing reductions of the incidence and mortality attributable to tuberculosis (TB), in 2014 there were an estimated 9.6 million new TB cases (12% of patients were coinfected with HIV-1) and 1.5 million deaths (27% of patients were coinfected with HIV-1) (1). In the case of rifampin-susceptible pulmonary tuberculosis, World Health Organization (WHO) guidelines advocate a daily regimen of 2 months of intensive-phase therapy with the first-line drugs rifampin, isoniazid, pyrazinamide, and ethambutol followed by 4 months of continuation-phase therapy with rifampin and isoniazid. Fixed-dose combination formulation (FDC) tablets are widely used to deliver standardized doses according to weight (2).

There are multiple causes leading to significant interindividual pharmacokinetic (PK) variability, including pharmacogenomics (3, 4), sex (5, 6), weight (6), and comorbidities, such as diabetes mellitus (5, 7–9). There have been contrasting findings published regarding the effect of HIV-1 coinfection on anti-TB drug pharmacokinetics, with some studies showing reduced drug exposures (10–12) and others showing no significant difference between HIV-1-coinfected and -uninfected patients (5, 13–15). Of note, the cohorts studied had various degrees of nutritional deprivation and immunosuppression and various proportions of patients concurrently taking antiretroviral therapy (ART). As other studies have shown contradictory findings on the role of plasma drug concentrations of anti-TB drugs, we thus wished to further address this in a contemporary cohort with good access to ART, resulting in many patients on ART and in HIV-1-coinfected patients generally being less advanced in terms of immunosuppression than the case in historical reports.

We hypothesized that there would be lower plasma drug concentrations of anti-TB drugs in HIV-1-coinfected patients than in HIV-1-uninfected patients. We aimed to assess pharmacokinetic parameters of rifampin, isoniazid, and pyrazinamide in a cohort with an adequate sample size by utilizing strong pharmacokinetic analysis methods to allow relatively accurate delineation and attribution of PK variability. Due to budgetary constraints, we focused on the three drugs with foremost importance in the anti-TB regimen (16).

The relationship between drug concentrations of standard-dose first-line anti-TB drugs and drug side effect profiles is unclear. Serious adverse drug reactions (common terminology criteria for adverse events [CTCAE] [17] grade 3 and above), such as drug-induced hepatotoxicity, are well documented and can sometimes be attributed to a specific anti-TB drug based upon temporality, cessation, and sequential drug rechallenge (18). Mild to moderate drug side effects of CTCAE grades 1 and 2 are common (19–21). Although these may be difficult to attribute to a specific anti-TB drug with any certainty, they are likely to contribute to suboptimal adherence (22) and may adversely affect treatment outcomes (19).

We also aimed to determine whether plasma drug concentrations were associated with reported drug side effects at 2 months.

## MATERIALS AND METHODS

### Patients.

Patients with GeneXpert MTB/RIF-confirmed rifampin-susceptible pulmonary TB were recruited at the Ubuntu HIV/TB Clinic, Site B, Khayelitsha, South Africa, as part of a prospective cohort study (Human Research Ethics Committee approval 568/2012) assessing the frequency and determinants of acquired drug resistance in a programmatic setting. A subcohort was invited to participate in a nested pharmacokinetic study between July 2013 and April 2014. All patients provided written consent prior to participation. Detailed sociodemographic data, past TB treatment history, and comorbidity data were collected. Participants underwent HIV testing (Abbott Architect HIV Ag/Ab Combo test), a CD4 lymphocyte count, and HIV-1 viral load quantification at baseline. Anti-TB drugs were delivered in a 4-drug FDC supplied by the National Tuberculosis Control Programme (Rifafour e-275 [Sanofi-Aventis] or Ritib [Aspen, South Africa]). Each tablet contained rifampin at 150 mg, isoniazid at 75 mg, pyrazinamide at 400 mg, and ethambutol at 275 mg. Weight band-based dosing was used in line with WHO guidelines (2). Patients weighing 38 to 55 kg, >55 to 70 kg, and >70 kg were given doses of 3, 4, and 5 tablets, respectively. Anti-TB drugs were administered 7 days/week, along with 25 mg pyridoxine. Clinical care remained the responsibility of the Site B TB clinic.

### Characterization of side effects.

Patients were interviewed at the 2-month clinical review by use of a systems-based symptom questionnaire that included the categories central nervous/neuropsychiatric, peripheral nervous, gastrointestinal, musculoskeletal, skin, and other. Attribution of causality to the anti-TB regimen was made in the “probable”/“possible” categories per the WHO-Uppsala Monitoring Center system (18).

### Pharmacokinetics.

Pharmacokinetic sampling was carried out for rifampin, isoniazid, and pyrazinamide after 7 to 8 weeks of anti-TB drugs. This time point was chosen to maximize applicability to a programmatic setting, i.e., a point of routine evaluation prior to switching from intensive-phase to continuation-phase therapy. In addition, this time point ensured that a majority of HIV-1-coinfected patients were prescribed ART and that rifampin autoinduction would be complete. Patients fasted for 8 h on the day of pharmacokinetic study and consumed standardized meals at 2 h and 4 to 5 h postdose. Blood samples were obtained immediately before (predose) and 1, 2, 3, 4, 6, and 8 h after drug ingestion. They were immediately placed on ice, and plasma was separated by centrifugation within 30 min before storage at −80°C until analysis. The storage tubes containing the plasma samples were transferred on dry ice to the Pharmacology Laboratory at the University of Cape Town, where drug concentrations were determined using validated liquid chromatography-tandem mass spectrometry (LC-MS/MS) methods. The methods were validated over the following concentration ranges: 0.0977 to 26.0 μg/ml for isoniazid, 0.117 to 30.0 μg/ml for rifampin, and 0.200 to 80.0 μg/ml for pyrazinamide (23). The % nominal concentration (accuracy) values were 99.2%, 98.1%, and 99.4% for rifampin, 100.5%, 100.1%, and 99.4% for isoniazid, and 100.8%, 103.7%, and 102.1% for pyrazinamide at the low, medium, and high quality control levels, respectively, during interday sample analysis. The precision (% coefficient of variation [% CV]) was less than 3% at the low, medium, and high quality control levels. Concentrations of rifampin, isoniazid, and pyrazinamide below the validation range of the assay were reported as “below the limit of quantification” (BLQ).

Plasma concentration-time data from all subjects were simultaneously analyzed by a nonlinear mixed-effects model by utilizing Monolix (version 4.3.3; Lixoft). Previously published structural models were selected and optimized with the current data (24–26). The structural models tested included one- and two-compartment dispositions with first-order elimination and first-order absorption, with the presence of an absorption lag time or a delay modeled via a chain of transit compartments. Interoccasional variability (IOV) was included by treating the drug concentration measured prior to the observed dose administration (predose concentration) as a separate pharmacokinetic occasion. Random interindividual variability (IIV) and IOV values were assumed to be lognormally distributed, and correlations between the random effects were tested. A mixture model was evaluated to explore the multimodal distribution of isoniazid clearance (CL) due to the polymorphic *N*-acetyltransferase-2 (NAT2) gene. Data points which were BLQ were treated as censored and handled with the Monolix implementation of the M3 method (27). Allometric scaling with either total body weight or fat-free mass (FFM) (28) was applied to CL and the volume of distribution (*V*) as suggested by Anderson and Holford (29). Fat-free mass was calculated using the empirical model developed by Janmahasatian et al. (30), as follows: FFM = (WHS_max_ × ht^2^ × wt)/(ht^2^ × WHS_50_ × wt), where “wt” denotes body weight in kilograms, “ht” denotes height in meters, and the constants WHS_max_ and WHS_50_ have values of 42.92 and 30.93, respectively, for men and 37.99 and 35.98, respectively, for women.

Other covariates tested included the effects of sex, age, serum albumin, total protein, HIV serostatus, CD4 lymphocyte count (as a binary variable, i.e., above or below 200 cells/mm^3^), type of ART (none versus nonnucleoside reverse transcriptase inhibitor [NNRTI]-based ART versus protease inhibitor [PI]-based ART), total dose, and dose (doses in milligrams per kilogram of body weight). Model development and selection were based on optimization of the objective function value (OFV), inspection of goodness-of-fit plots, including visual predictive checks (*n* = 500), and biological plausibility. Stepwise covariate selection was performed using a drop in OFV of >3.84 (corresponding to a significance level of 5%) as the cutoff for inclusion and an increase of >6.63 OFV points as a cutoff for the backward elimination step. The OFV was obtained using importance sampling (*n* = 20,000), and the precision of the parameter estimates was obtained using a stochastic approximation based on the Fisher information matrix.

Finally, model-based individual pharmacokinetic parameter values referring to the pharmacokinetic profile after the observed dose were used in the R package Simulx (31) to simulate steady-state individual profiles and to calculate the peak concentration (*C*_max_) and the area under the concentration-time curve from 0 to 24 h (AUC_0–24_).

### Statistical analyses.

The Wilcoxon-Mann-Whitney test was used to compare PK exposures as those with side effects and those without. Logistic regression analyses were used to calculate odds ratios (ORs) for side effects at different drug exposure quartiles, and the ORs were adjusted for potential confounders. Stata, version 13.1 (College Station, TX), and GraphPad Prism 6.0 (La Jolla, CA) were used for all analyses.

## RESULTS

### Patient demographics.

Of the 100 study participants, 57% were male and 65% were coinfected with HIV-1, with a median CD4 lymphocyte count of 233 cells/mm^3^ (interquartile range [IQR], 106 to 386 cells/mm^3^). Among HIV-1-coinfected patients, the proportion on ART increased from 27/65 (42%) patients at baseline to 50/65 (77%) patients at the time of pharmacokinetic study, with 45/50 (90%) patients on NNRTI-based (96% on efavirenz and 5% on nevirapine) regimens and 5/50 (10%) patients on PI-based (lopinavir/ritonavir [LPV/r]) regimens.

The median (IQR) body mass index (BMI) and age were 21 kg/m^2^ (19 to 23 kg/m^2^) and 33 years (29 to 40 years), respectively. Table 1 provides the clinical characteristics of the pharmacokinetic cohort, stratified by HIV-1 serostatus.

**TABLE 1:** Clinical characteristics of the cohort, stratified by HIV-1 serostatus

Clinical characteristic^*a*^	Value		
	Whole PK cohort (*n* = 100)	HIV-1-infected patients (*n* = 65)	HIV-1-uninfected patients (*n* = 35)
Male (no. [%])	57 (57)	30 (46)	27 (77)
Xhosa ethnicity (no. [%])	98 (98)	63 (97)	35 (100)
Median (IQR) age (yr)	33 (29–40)	34 (30–40)	32 (27–38)
No. (%) of patients with smoking history			
Current	24 (24)	9 (14)	15 (43)
Previous	27 (27)	19 (29)	8 (23)
Never	49 (49)	37 (57)	12 (34)
No. (%) of patients with characteristic			
Alcohol consumption	37 (37)	16 (25)	17 (49)
Recreational drug use	5 (5)	3 (5)	2 (6)
Previously in prison	14 (14)	10 (15)	4 (11)
Previous mining history	5 (5)	1 (1)	4 (11)
Retreatment	39 (39)	29 (45)	10 (29)
Type 2 diabetes mellitus	4 (4)	3 (5)	1 (3)
Median (IQR) BMI at baseline (kg/m^2^)	21 (19–23)	21 (20–23)	20 (19–23)
Median (IQR) BMI at PK study (kg/m^2^)	21.5 (20–23)	22 (20–23)	20.5 (19–23)
Median (IQR) FFM at PK study (kg)	45 (38–49)	40.5 (36–47)	49 (46–51)
Median (IQR) CD4 count (cells/mm^3^)		233 (106–386)	
No. (%) with viral load of <40 copies/ml at baseline		17 (26)	
Median (IQR) albumin concn at PK study (g/liter)	38 (34–40)	36 (34–39)	38 (40–43)
Median (IQR) total protein concn at PK study (g/liter)	86 (79–92)	88 (82–93)	82 (75–86)
Median (IQR) time on ART at time of PK study (mo)		1.32 (0–15.5)	
No. (%) of patients with smear grade at baseline			
3+	24 (24)	14 (21.5)	10 (29)
2+	22 (22)	11 (17)	11 (31)
1+	20 (20)	14 (21.5)	6 (17)
Scanty/negative	34 (34)	26 (40)	8 (23)
Median (IQR) baseline time to culture positivity (days)	10 (7–14)	12 (7–15)	8 (6.5–12.5)
No. (%) of patients with extensive radiological disease at baseline	71 (71)	41 (63)	30 (86)
No. (%) of patients with cavities at baseline	52 (52)	32 (49)	20 (57)
Median (range) dose at PK study (mg/kg)			
Rifampin	10 (7–11.5)	10 (7–11.5)	10 (7–11.5)
Isoniazid	5 (3.5–6)	5 (4–6)	5 (3.5–6)
Pyrazinamide	26 (19–31)	26 (20–31)	25.5 (19–31)
No. (%) of patients with side effects of TB treatment	35 (35)	25 (38)	10 (29)
No. (%) of poorly adherent patients per pill counts/self-reports at 2-month review	10 (10)	8 (12)	2 (6)

a Characteristics are reported for the time of diagnosis (baseline) unless otherwise specified (at PK visit or 2-month visit). Abbreviations: BMI, body mass index; FFM, fat-free mass; ART, antiretroviral therapy; PK, pharmacokinetics.

### Patient pharmacokinetic parameters.

**FIG 1:**
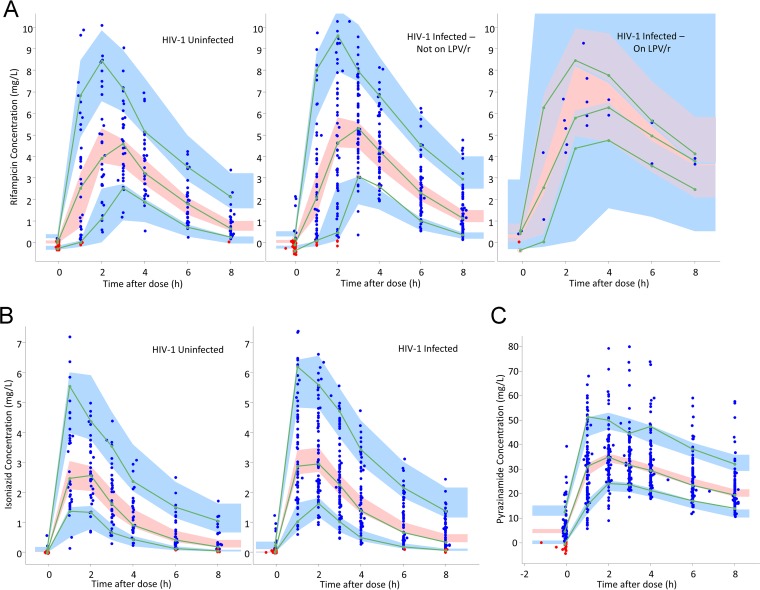
(A) Visual predictive check for rifampin concentration versus time, stratified by HIV-1 serostatus and coadministration of lopinavir/ritonavir (LPV/r). The blue dots are observed concentrations, and the red dots are simulation-based values below the limit of quantification (BLQ). Lines are 10th, 50th, and 90th percentiles for observed data, while the shaded areas represent the 90% confidence intervals for the same percentiles, as predicted by the model. (B) Visual predictive check for isoniazid concentration versus time, stratified by HIV-1 serostatus. The blue dots are observed concentrations, and the red dots are simulation-based values below the limit of quantification (BLQ). Lines are 10th, 50th, and 90th percentiles for observed data, while the shaded areas represent the 90% confidence intervals for the same percentiles, as predicted by the model. (C) Visual predictive check for pyrazinamide concentration versus time. The blue dots are observed concentrations, and the red dots are simulation-based values below the limit of quantification (BLQ). Lines are 10th, 50th, and 90th percentiles for observed data, while the shaded areas represent the 90% confidence intervals for the same percentiles, as predicted by the model.

The final population pharmacokinetic parameter estimates for rifampin, isoniazid, and pyrazinamide are shown in Tables 2 to 4, including the precision of parameter estimates and shrinkage values for the random effects (32). Visual predictive checks are provided in Fig. 1.

**TABLE 2:** Parameter values estimated by the final pharmacokinetic model for rifampin^*a*^

Parameter	Estimated typical value (95% CI)	% variability (95% CI), shrinkage^*b*^	
		Interoccasional	Interindividual
Bioavailability (*F*)	1 (fixed)	29.1 (24.2–34.0), 20	
Absorption lag time (h)	0.691 (0.590–0.791)	76.2 (62.0–89.9), 24	
Absorption constant (h^−1^)	1.21 (1.03–1.38)	63.2 (49.0–77.5), 26	
CL/*F* (liters/h)^*c*^			
HIV-1-uninfected patients	25.1 (21.8–28.4)		34.3 (28.8–39.8), 10
HIV-1-infected patients not on LPV/r	19.9 (17.8–21.8)		34.3 (28.8–39.8), 10
HIV-1-infected patients on LPV/r	10.8 (7.08–14.5)		34.3 (28.8–39.8), 10
Vol of distribution (liters)^*c*^	56.4 (53.7–59.1)		
Additive error (mg/liter)	0.196 (0.174–0.218)		
Proportional error (%)	15.0 (13.2–16.8)		

a Abbreviations: *F*, bioavailability; LPV/r, lopinavir/ritonavir; 95% CI, 95% confidence interval.

b Interindividual and -occasional variabilities were assumed to be lognormally distributed and are reported here as approximate % CV. For interoccasional variability terms, the shrinkage is reported only for the occasion with intensive sampling (not the predose).

c Clearance and volume of distribution were allometrically scaled using individual values for fat-free mass (FFM), so the typical values reported here refer to the median value for FFM in the cohort, i.e., 45 kg (e.g., a 1.7-m tall man weighing 51 kg).

**TABLE 3:** Parameter values estimated by the final pharmacokinetic model for isoniazid

Parameter	Estimated mean population value (95% CI)	% variability (95% CI), shrinkage^*a*^	
		Interoccasional	Interindividual
Bioavailability (*F*)	1 (fixed)	32.3 (27.2–37.4), 12	
Mean transit time (h)	0.32 (0.12–0.51)	92.7 (65.3–120), 35	
No. of absorption transit compartments	2.04 (1.55–2.53)		
Absorption constant (h^−1^)	1.20 (1.03–1.36)	17.7 (11.8–23.6), 60	
CL/*F* (liters/h)^*b*^			
HIV-1-uninfected patients	26.0 (21.1–30.9)		54.8 (46.9–62.6), 3
HIV-1-infected patients	20.02 (12.9–25.2)		54.8 (46.9–62.6), 3
Vol of distribution of central compartment (liters)^*b*^	31.9 (30.8–36.2)		
Intercompartmental CL/*F* (liters/h)^*b*^	12.6 (6.13–19.07)		
Vol of distribution of peripheral compartment (liters)^*b*^	21.4 (18.5–24.4)		
Additive error (mg/liter)	0.0146 (0.003–0.03)		
Proportional error (%)	13.1 (11.9–14.3)		

a Interindividual and -occasional variabilities were assumed to be lognormally distributed and are reported here as approximate % CV. For interoccasional variability terms, the shrinkage is reported only for the occasion with intensive sampling (not the predose).

b All clearance and volume parameters were allometrically scaled using individual values for fat-free mass (FFM), so the typical values reported here refer to the median value for FFM in the cohort, i.e., 45 kg (e.g., a 1.7-m tall man weighing 51 kg).

**TABLE 4:** Parameter values estimated by the final pharmacokinetic model for pyrazinamide

Parameter	Estimated population value (95% CI)	% variability (95% CI), shrinkage^*a*^	
		Interoccasional	Interindividual
Bioavailability (*F*)	1 (fixed)	13.1 (10.2–16), 31	
Mean transit time (h)	0.74 (0.65–0.84)	54.5 (45.1–63.9), 19	
No. of absorption transit compartments	2.06 (1.59–2.53)		
Absorption rate constant (h^−1^)	50.0 (fixed)^*c*^		
Vol of distribution (liters)^*b*^	41.9 (40.4–43.4)		
CL/*F* (liters/h)^*b*^	4.17 (3.90–4.44)		29.6 (24.7–34.5), 8
Additive error (mg/liter)	1.95 (1.77–2.13)		
Proportional error (%)	10.7 (9.60–11.80)		

a Interindividual and -occasional variabilities were assumed to be lognormally distributed and are reported here as approximate % CV. For interoccasional variability terms, the shrinkage is reported only for the occasion with intensive sampling (not the predose).

b All clearance and volume parameters were allometrically scaled using individual values for fat-free mass (FFM), so the typical values reported here refer to the median value for FFM in the cohort, i.e., 45 kg (e.g., a 1.7-m tall man weighing 51 kg).

c The model estimated a very large value for the absorption constant, so it was fixed to 50 to stabilize the model without significantly affecting the fit.

The optimized structural model for rifampin was a one-compartment model with first-order elimination and first-order absorption, with an absorption lag time. A two-compartment model with first-order elimination and absorption through a series of transit compartments was optimal for isoniazid. Finally, a one-compartment model with first-order elimination and transit compartment absorption was used for pyrazinamide. FFM was found to be the most suitable body size descriptor for allometric scaling of all CL and *V* parameters, and it improved the OFV by 20, 34, and 64 points for rifampin, isoniazid, and pyrazinamide, respectively.

Since HIV-1 serostatus (infected versus not infected) and ART status (on ART versus not on ART) are colinear, the effects of the covariates HIV-1 status and ART status on the pharmacokinetic parameters were tested separately. HIV-1 status as a covariate caused improvement in the model to a greater extent than ART status did (7-point versus 4-point drop in OFV), and diagnostic plots were better for the model including HIV-1 status; hence, this was selected in the final model rather than ART status. The models found that HIV-1 coinfection significantly decreased the CL of rifampin (21% decrease; OFV drop of 7.00 points; *P* < 0.01) (Table 2) and isoniazid (23% decrease; OFV drop of 8.63 points; *P* < 0.01) (Table 3). Inclusion of HIV-1 coinfection in the covariate model did not significantly improve the model fit for pyrazinamide. Although HIV-1-infected participants had lower FFM than HIV-1-uninfected participants (Table 1), the effect of HIV-1 on CL was independent of differences in FFM.

We split the covariate ART status into types of ART (i.e., no ART, NNRTI-based ART, and LPV/r-based ART) and tested these as further covariates (in addition to the HIV effect). We separately tested the effects of both NNRTI-based regimens (yes/no) and LPV/r-based regimens (yes/no) as covariates on bioavailability, *V*, and CL in an optimized model which was already adjusted for the effect of HIV-1 status on CL. On top of the effect of HIV-1 on CL, patients on a double-dose LPV/r-based ART regimen (dosed at 800 mg/200 mg twice daily in all 5 cases) had a further significant decrease in rifampin CL of 46% (OFV drop of 7.00 points; *P* < 0.01), and hence they had an increased AUC_0–24_ (Table 2). When the model was rerun with exclusion of the 5 participants on PI-based regimens, the effect of HIV-1 on CL was still significant.

During model development, the multimodal distribution of isoniazid CL attributed to the polymorphic nature of the NAT2 genotype (3) was described using a mixture model which improved the model fit. However, the current version of Monolix does not support both mixture modeling and estimation of interoccasional variability, which was used to describe variability in the predose sample, so the latter was included because it was more significant in terms of model fit.

**FIG 2:**
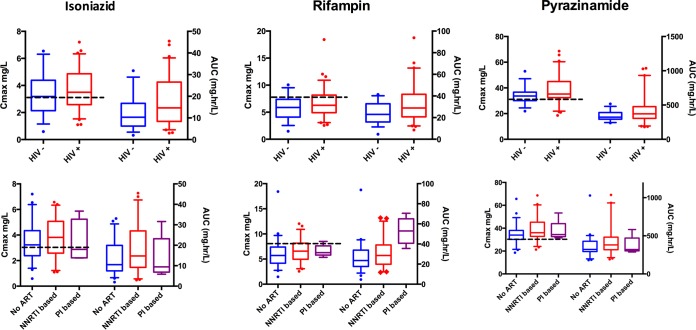
Pharmacokinetic measures *C*_max_ and AUC_0–24_, stratified by HIV serostatus and antiretroviral therapy regimen. The box-and-whisker plots show model-derived PK measures. *C*_max_ and AUC_0–24_ are plotted on the left and right *y* axes, respectively. The boxes show medians and interquartile ranges. The whiskers represent the 5th to 95th percentiles and illustrate the variability in both HIV-1-infected (+) and HIV-1-uninfected (−) patients and different antiretroviral therapy categories. Patients on inhibitors appeared to have higher rifampin AUC_0–24_ values than patients in the no-ART and NNRTI-based categories. The dotted black lines indicate the current recommended thresholds for *C*_max_ of 3 mg/liter, 8 mg/liter, and 30 mg/liter for isoniazid, rifampin, and pyrazinamide, respectively. No tests for statistical significance were run to generate *P* values for these *post hoc* individual estimates, as the reported individual values are based on the population PK models and are hence interdependent. The significance of the respective covariate effects (Tables 2 to 4) was tested within the model. Abbreviations: ART, antiretroviral therapy; NNRTI, nonnucleoside reverse transcriptase inhibitor; PI, protease inhibitor; *C*_max_, maximum concentration; AUC, area under the curve from 0 to 24 h.

Figure 2 shows *C*_max_ and AUC_0–24_ values stratified by HIV-1 serostatus. For all 3 drugs, either pharmacokinetic exposures were increased in those infected with HIV-1 or no difference was detected. There were no differences in exposures between HIV-1-infected patients with CD4 lymphocyte counts above and below 200 cells/mm^3^. Among HIV-1-infected participants, 41% had a low isoniazid *C*_max_ (<3 mg/liter), 75% had a low rifampin *C*_max_ (<8 mg/liter), and 31% had a low pyrazinamide *C*_max_ (<35 mg/liter) (33). Among HIV-1-uninfected participants, 46% had a low isoniazid *C*_max_, 88% had a low rifampin *C*_max_, and 63% had a low pyrazinamide *C*_max_.

**FIG 3:**
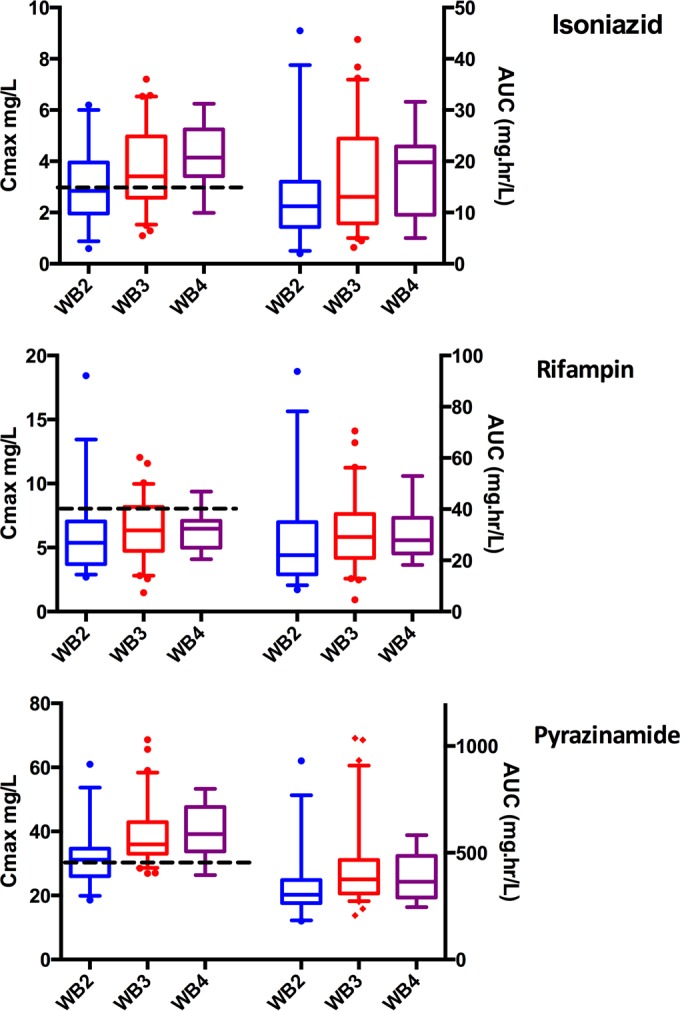
Pharmacokinetic measures *C*_max_ and AUC_0–24_, stratified by WHO weight band. The box-and-whisker plots show model-derived PK measures stratified by WHO weight band. *C*_max_ and AUC_0–24_ values are plotted on the left and right *y* axes, respectively. The boxes show medians and interquartile ranges. The whiskers represent the 5th to 95th percentiles. The predictions include allometric scaling, which is necessary to account for nonlinear differences by weight/size. This explains differences in PK measures despite the same dosing (milligram per kilogram of body weight) by weight band. The dotted black lines indicate the current recommended thresholds for *C*_max_ of 3 mg/liter, 8 mg/liter, and 30 mg/liter for isoniazid, rifampin, and pyrazinamide, respectively. Abbreviations: *C*_max_, maximum concentration; AUC, area under the curve from 0 to 24 h; WB, weight band.

The ranges of doses (in milligrams per kilogram of body weight) for the three drugs are shown in Table 1. Eight participants required a change in weight band during treatment. Three had their dose adjusted appropriately, one was put in a weight band higher than his weight, and four were put in a weight band lower than their weight. Hence, on the day of pharmacokinetic sampling, 95 participants were being dosed correctly according to current weight and height bands. Patients in the lowest weight band had lower drug exposures, and this was explained by a relatively higher CL in smaller individuals. Figure 3 shows differences in pharmacokinetic exposures stratified by the WHO weight band doses assigned by the program. The predictions shown included allometric scaling with FFM, which accounted for the increased clearance per kilogram of body weight in smaller individuals and resulted in a significant improvement of the model.

**FIG 4:**
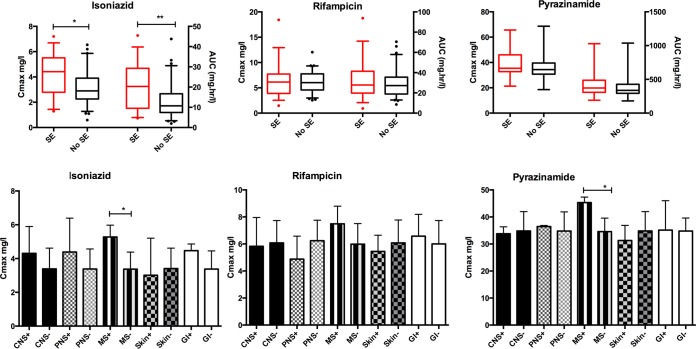
Pharmacokinetic measures *C*_max_ and AUC_0–24_, stratified by side effect profile. The top 3 graphs show *C*_max_ values on the left axis and AUC_0–24_ values on the right *y* axis, stratified by the presence or absence of drug side effects. The bottom 3 graphs detail drug peak concentrations in those with (+) and without (−) CNS, PNS, MS, skin, and GI side effects. Abbreviations: *C*_max_, peak concentration; AUC, area under the curve from 0 to 24 h; SE, side effects; CNS, central nervous system; PNS, peripheral nervous system; MS, musculoskeletal system; GI, gastrointestinal.

All side effects were of CTCAE grades 1 and 2 and did not require drug withdrawal. Thirty-five participants (35%) reported CTCAE grade 1 and 2 side effects which were possibly/probably attributed to anti-TB drugs, including central nervous system/neuropsychiatric (4/35 patients), peripheral neuropathy (7/35 patients), nausea/gastrointestinal (11/35 patients), musculoskeletal (11/35 patients), skin (8/35 patients), and other (2/35 patients) effects. Patients presenting side effects had a significantly higher median *C*_max_ for isoniazid (4.42 mg/liter [IQR, 2.79 to 5.51 mg/liter]) than those who did not (2.89 mg/liter [IQR, 2.28 to 3.87 mg/liter]) (*P* = 0.001). After adjustments for age, HIV-1 serostatus, diabetes mellitus status, alcohol intake, and previous isoniazid treatment, the highest quartile of isoniazid AUC_0–24_ values was still associated with increased side effects (OR, 7.11 [95% confidence interval, 1.99 to 25.47]; *P* = 0.003) compared to the lowest quartile of isoniazid AUC_0–24_ values (Table 5). There were no significant differences for rifampin or pyrazinamide (Fig. 4). Although there was a trend of high isoniazid *C*_max_ values for those with central nervous system, peripheral nervous system, gastrointestinal, and musculoskeletal side effects, this was statistically significant only for musculoskeletal side effects. A significantly higher pyrazinamide *C*_max_ was also seen for patients with musculoskeletal side effects (Fig. 4).

**TABLE 5:** Risk factors for reported side effects at 2-month review by univariate and multivariate models

Variable^*a*^	OR (95% CI) for reported side effects	Adjusted OR (95% CI) for reported side effects
INH AUC_0–24_ quartile 1	1	1
INH AUC_0–24_ quartile 2	1.26 (0.34–4.84)	1.19 (0.29–4.87)
INH AUC_0–24_ quartile 3	1.88 (0.52–6.84)	2.08 (0.54–8.07)
INH AUC_0–24_ quartile 4	7.11 (1.99–25.47)	9.12 (2.28–36.55)
10-yr increase in age	1.09 (0.71–1.67)	1.14 (0.68–1.92)
Previous isoniazid treatment	1.54 (0.67–3.55)	1.76 (0.63–4.97)
HIV-1 serostatus	1.56 (0.64–3.80)	1.12 (0.41–3.08)
Alcohol intake	1.46 (0.63–3.41)	1.92 (0.72–5.16)
Type 2 diabetes mellitus	1.99 (0.26–14.17)	1.67 (0.12–14.28)

a Abbreviations: INH, isoniazid; AUC_0–24_, area under the concentration-time curve from 0 to 24 h.

## DISCUSSION

Peak concentrations and AUC_0–24_ values for rifampin, isoniazid, and pyrazinamide were low and highly variable, and the findings were comparable to those for other cohorts in both similar (southern African) (6, 10, 13, 34, 35) and different (36–38) study populations. There have been previous studies examining anti-TB pharmacokinetics in HIV-1-coinfected patients and an HIV-1-uninfected comparator group. However, none of these studies included HIV-1-coinfected patients concomitantly taking ART. Reduced rifampin concentrations have been recorded for HIV-1-coinfected patients with diarrhea, and this has been associated with malabsorption and advanced immunosuppression (11, 39, 40). Further studies have also shown evidence of reduced rifampin concentrations in HIV-1-coinfected patients (10, 12), while others showed no significant difference (5, 13, 15, 41). There have also been contrasting results in the literature regarding the effect of HIV-1 coinfection on the pyrazinamide concentration (10, 42).

In this cohort of ambulatory patients, with 77% of HIV-1-coinfected patients on ART, there was no evidence of lower plasma concentrations of rifampin, isoniazid, or pyrazinamide in HIV-1-coinfected patients. Conversely, the population pharmacokinetic model which accounted for the effect of FFM showed evidence of reduced rifampin and isoniazid CL in HIV-1-coinfected compared to HIV-1-uninfected patients, leading to increased AUC_0–24_ values.

Although the use of ART or CD4 stratification as a covariate did not significantly improve the model, there was an independent effect of LPV/r versus no LPV/r on the rifampin AUC_0–24_, which again was explained by a reduction in CL. Although only 5 patients in the study were on an ART regimen inclusive of LPV/r, the effect of double-dose LPV/r on rifampin exposures was statistically significant. This has not been reported previously. Rifampin is a substrate of p-glycoprotein, organic anion-transporting polypeptide 1B1 (OATP1B1), and OATP1B3, which are involved in its transporter-mediated efflux in the liver, and hence in biliary clearance. Lopinavir and ritonavir are inhibitors of both p-glycoprotein and OATP1/3 (43). Hence, this may be a potential mechanism for decreased clearance of rifampin. Inhibition of gastrointestinal p-glycoprotein may also increase systemic rifampin concentrations. There are potential implications for dosing and toxicity profiles, particularly in future regimens incorporating higher doses (per kilogram) of rifampin, and these findings should be explored in further pharmacokinetic studies. These results provide evidence that at the time of switch to the continuation phase, ambulant HIV-1-coinfected patients undergoing immune reconstitution on ART do not have reduced anti-TB drug concentrations compared to HIV-1-uninfected patients. These findings are not necessarily generalizable to HIV-1-coinfected patients in an inpatient setting or to those with advanced immunosuppression (the median CD4 count of this cohort was 233 cells/mm^3^).

As previously reported (6), weight, and in particular FFM, influenced CL in a nonlinear fashion, and hence uniform dosing (in milligram per kilogram of body weight) across the WHO weight bands was associated with the lowest weight band having a trend of lower drug concentrations than those with the highest weight band. Therefore, dosing could be optimized according to FFM, and in particular, dosing for the lower weight band should be reviewed.

Having adjusted for potential confounders, we still found a significantly increased proportion of side effects in patients with isoniazid AUC_0–24_ values in the highest quartile. Overall incidences of drug side effects secondary to isoniazid reported in the literature range from 1 to 3% for dermatological, gastrointestinal, and neurological side effects and from 1 to 17% for hypersensitivity reactions (20). Thirteen of the 16 patients who had side effects and were in the highest isoniazid AUC_0–24_ quartile were coinfected with HIV-1. Studies have shown that 8 to 20% of patients taking isoniazid can develop antinuclear antibodies. This is increased in slow acetylators (44) and may be potentiated in HIV-1 coinfection. One randomized controlled clinical trial conducted in Japan showed that isoniazid-related liver injury in the first 8 weeks of anti-TB treatment occurred in 78% of slow acetylators given standard 5-mg/kg doses, compared to 0% of slow acetylators given 2.5-mg/kg doses (45). Hence, significant pharmacokinetic variability for isoniazid, even with standard dosing, may contribute to toxicity.

There were several limitations in this study. Pharmacogenomic data, such as NAT2 and SLCO1B1 genotypes, were not available for incorporation into the population PK models. Drug concentration sampling was not repeated at different times during treatment and hence may have under- or overestimated the IOV secondary to changes in weight and immune reconstitution secondary to ART. A previous study of HIV-1-coinfected patients did not find an independent effect on TB pharmacokinetics for first-dose ART or steady-state ART (at 2 weeks) compared to day 1 of anti-TB drugs (6). There was no routine monitoring of blood tests, such as liver and renal function tests. Hence, asymptomatic adverse drug reactions would not have been ascertained.

In this outpatient setting with a high burden of HIV-1-coinfected patients, the majority of whom were undergoing immune reconstitution on ART, there was no evidence that HIV-1 coinfection led to lower anti-TB drug concentrations.
